# Blood flow velocity and thickness of the choroid in a patient with chorioretinopathy associated with ocular blunt trauma

**DOI:** 10.1186/s12886-017-0480-9

**Published:** 2017-06-08

**Authors:** Yuri Ishikawa, Yuki Hashimoto, Wataru Saito, Ryo Ando, Susumu Ishida

**Affiliations:** 1 Department of Ophthalmology, Faculty of Medicine and Graduate School of Medicine, Hokkaido University, Nishi 7, Kita 15, Kita-ku, Sapporo, 060-8638 Japan; 2 Kaimeido Eye and Dental Clinic, Sapporo, Japan

**Keywords:** Choroidal blood flow velocity, Choroidal thickness, Mean blur rate, Ocular blunt trauma

## Abstract

**Background:**

Choroidal circulation hemodynamics in eyes with ocular blunt trauma has not been quantitatively examined yet. We quantitatively examined changes in choroidal blood flow velocity and thickness at the lesion site using laser speckle flowgraphy (LSFG) and enhanced depth imaging optical coherence tomography (EDI-OCT) in a patient with chorioretinopathy associated with ocular blunt trauma.

**Case presentation:**

A 13-year-old boy developed a chorioretinal lesion with pigmentation extending from the optic disc to the superotemporal side in the right eye after ocular blunt trauma. The patient’s best-corrected visual acuity (BCVA) was 0.2 in the right eye. Indocyanine green angiography showed hypofluorescence from the initial phase, with a decrease of mean blur rate (MBR) on LSFG color map, which corresponded to the chorioretinal lesion. The BCVA and foveal outer retinal morphologic abnormality spontaneously improved during follow-up. MBR and choroidal thickness increased by 23–31% and 13–17 μm at the lesion site and by 11–22% and 33–42 μm at the fovea, respectively, during the 6-month follow-up period after baseline measurements in the affected eye. In contrast, these parameters showed little or no changes at the normal retinal site in the affected eye and the fovea in the fellow eye.

**Conclusions:**

Current data revealed that both blood flow velocity and thickness in the choroid at the lesion site decreased in the acute stage and subsequently increased together with improvements in visual function and outer retinal morphology. These results suggest that LSFG and EDI-OCT may be useful indices that can noninvasively evaluate activity of choroidal involvement in ocular blunt trauma-associated chorioretinopathy.

## Background

Ocular blunt trauma can cause retinal opacity in the macula and/or mid-periphery, which is known as commotio retinae [1, 2]. Visual impairment associated with commotio retinae often recover and most patients have favorable visual outcomes [3, 4]. However, cases due to severe injuries may develop permanent retinal degeneration following retinal opacity and have poor visual prognosis [5, 6]. Retinal degeneration is sometimes seen as a triangular shape at various sites of the fundus [7, 8]. Experimental studies of blunt trauma to the eye showed damage to photoreceptors and the retinal pigment epithelium (RPE), which regenerated during follow-up [9, 10]. Moreover, spectral domain optical coherence tomography (OCT) revealed outer retinal morphological impairment of various degrees corresponding to the sites with retinal opacities [4, 6] and the degree correlated with a poorer final visual outcome [3]. These observations suggest that outer retinal damage causes visual impairment in this disease, and the extent correlates with the level of visual impairment.

An experimental study with plastic casts showed a defect in the short posterior ciliary artery at the choroidal outer layer beneath the sites with retinal opacities following blunt trauma [10]. This suggests that disruption to the choroidal circulation occurs following breakdown of the short posterior ciliary arteries and may play a role in the pathogenesis of outer retinal damage secondary to ocular blunt trauma. Moreover, in both animal and human studies, indocyanine green angiography (ICGA) showed a defect in choroidal inflow or spasm of the choroidal artery corresponding to the lesion site [8, 11]. Therefore, ICGA is a useful tool that can evaluate various choroidal circulation disturbances in patients with the ocular blunt trauma [8]. However, choroidal circulation hemodynamics in chorioretinopathy with ocular blunt trauma has not been examined quantitatively, because ICGA is a qualitative examination.

Laser speckle flowgraphy (LSFG) has been used to investigate ocular blood flow velocity quantitatively. LSFG targets moving red blood cells with a diode laser (wavelength, 830 nm) to illuminate the ocular fundus and reflected light from moving erythrocytes produces blurring within the speckle pattern [12]. The mean blur rate (MBR), automatically calculated from variations in the degree of blurring, is a quantitative index of the relative blood flow velocity [13] with high reproducibility of measurements [14]. As the MBR originates mainly from the choroid, the value reflects choroidal hemodynamics, particularly at the macula [15]. Therefore, LSFG is a suitable device to monitor changes in choroidal circulation hemodynamics during the course of various chorioretinal diseases [16–18].

We used LSFG and enhanced depth imaging (EDI)-OCT to quantitatively investigate the time course of changes in circulation hemodynamics and thickness of the choroid in a patient with chorioretinopathy associated with ocular blunt trauma.

## Case presentation

A 13-year-old boy complained of blurred vision in his right eye after trauma from a soccer ball. The patient was referred to our hospital 21 days after the injury. The patient’s medical and family histories were unremarkable.

**Fig. 1:**
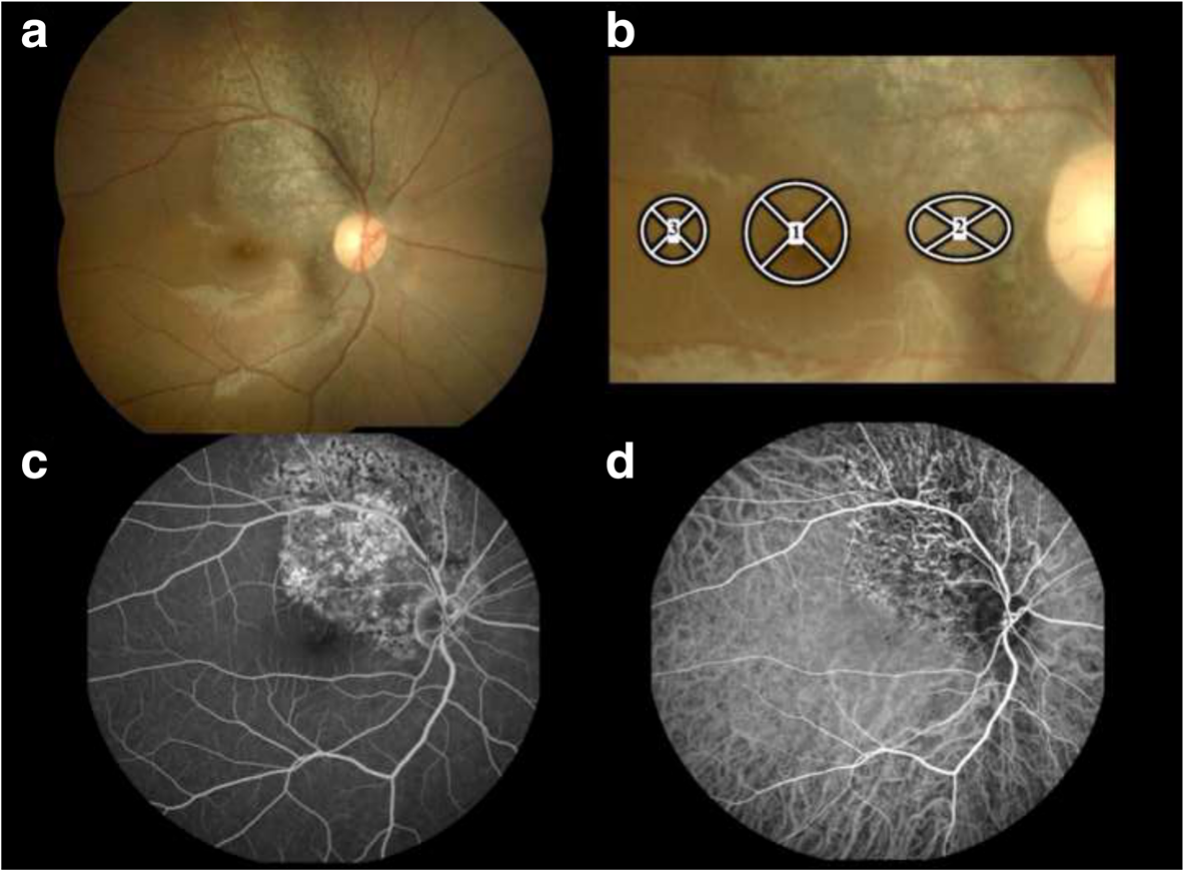
Photographs of the right eye at the initial visit in a patient with chorioretinopathy associated with ocular blunt trauma. **a** Fundus photograph showing a fan-shaped grayish chorioretinal lesion with pigmentation extending to the superotemporal side from the optic disc and depigmentation at the fovea. **b** A magnified view of A. Circles represent the same sites as Circles 1–3 set on laser speckle flowgraphy (LSFG) color map in Fig. 2b and d. **c** Late-phase fluorescein angiography showing a window defect corresponding to the chorioretinal lesion. **d** Venous-phase indocyanine green angiography showing hypofluorescence corresponding to the lesion site. Narrowed middle or large choroidal vessels can be visualized within the hypofluorescence

**Fig. 2:**
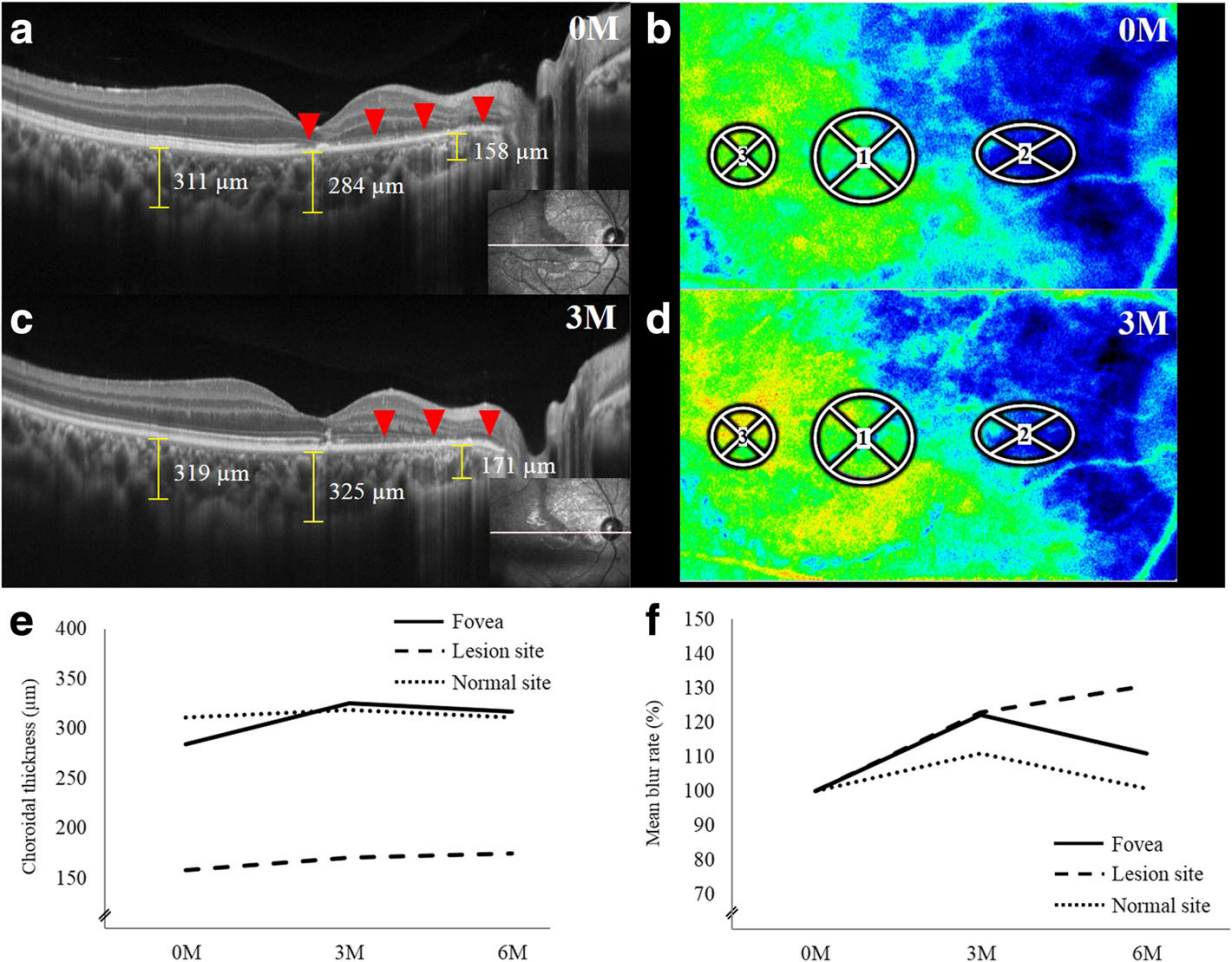
Images of enhanced depth imaging optical coherence tomography (**a**, **c**) and LSFG color map (**b**, **d**), and changes in choroidal thickness (**e**) and mean blur rate (MBR) (**f**) during follow-up in an eye with ocular blunt trauma-associated chorioretinopathy. **a**, **c**: At the initial visit, a horizontal image through the fovea shows loss of the ellipsoid zone corresponding to the fovea and the lesion site (**a**, *arrowheads*). The choroidal thickness examined at the same sites as Circles 1–3 set in Fig. 1b were 284, 158, and 311 um, respectively (**a**). Three months after the initial visit, impaired ellipsoid zone at the fovea improved, however it remained unchanged at the lesion area (**c**, *arrowheads*). The choroidal thickness increased at the fovea by 42 μm and at the lesion site by 13 μm, respectively. Meanwhile, it remained unchanged at the normal retinal site (+ 8 μm). **b**, **d**: On the LSFG color map, Circles 1–3 were set at the fovea, the lesion site, and the normal retinal site, respectively (**b**, **d**). The area of the color map is identical to Fig. 1b. At the initial visit, a decrease in the MBR was clearly visualized corresponding to the chorioretinal lesion (**b**). The *blue color* indicates a low MBR, while the *red color* shows a high MBR. Three months after the initial visit, the MBR increased at the fovea and the lesion site (**d**). **e**, An increase of 33–42 μm and 13–17 μm were detected at the fovea and the lesion site, respectively, during the 6-month follow-up period. In contrast, there were little or no changes in Circle 3 (normal site, 0–8 μm). **f**, When compared with the baseline MBR (100%), an increase of 11–22 and 23–31% were detected in Circles 1 (fovea) and 2 (lesion site), respectively, during the 6-month follow-up period. In contrast, there were little or no changes in Circle 3 (normal site, 1–11%)

At the initial visit, the patient’s best-corrected visual acuity (BCVA) was 0.2 OD and 1.5 OS, and the intraocular pressure (IOP) was 16 mmHg OD and 19 mmHg OS. His left eye was normal. Slit-lamp examination revealed no abnormal findings in the anterior segment and lens OD. Funduscopy showed a fan-shaped grayish chorioretinal lesion with pigmentation extending from the optic disc to the superotemporal side OD (Fig. 1a, b). The fovea demonstrated depigmentation of the RPE. Fluorescein angiography showed choroidal filling delay in the initial phase and a window defect in the late phase (Fig. 1c), corresponding with the chorioretinal lesion. ICGA showed hypofluorescence from the initial to the late phase corresponding with the lesion (Fig. 1d). Narrowed middle or large choroidal vessels could be observed within the hypofluorescence. EDI-OCT image showed the loss of the ellipsoid zone and interdigitation zone at the fovea and the lesion area (Fig. 2a, arrowheads). The patient was diagnosed with chorioretinopathy associated with ocular blunt trauma OD and was followed up with no treatment. Three months after the initial visit, the BCVA increased to 0.6 OD and the ellipsoid zone at the fovea improved, although it remained unchanged at the lesion area (Fig. 2c). Eleven months after the initial visit, his BCVA, funduscopic, and OCT findings remained unchanged compared with those at 3 months. Twenty-one months after the initial visit, his BCVA was 0.7 OD, with improvement at both the ellipsoid zone and interdigitation zone at the fovea.

In order to quantitatively examine choroidal blood flow velocity in the present case, LSFG measurements using LSFG-NAVI (Softcare, Fukuoka, Japan) were obtained five consecutive times for the affected eye at the initial visit and 3 and 6 months after the initial visit and the fellow eye at the initial visit and 11 months after the initial visit. The current study was approved by the ethics committee of Hokkaido University Hospital. Informed consent was obtained after an explanation of the nature and possible consequences of the study, which followed the standard of care outlined in the Declaration of Helsinki. The pupils were dilated with 0.5% tropicamide and 0.5% phenylephrine hydrochloride 20 min prior to LSFG testing. During each measurement, eye movement and focus adjustment were monitored using live-capture images. To evaluate the change in relative blood flow velocity at various retinal sites in the affected eye, measurement circles were set on the LSFG color map as follows: Circle 1 at the fovea; Circle 2 at a lesion site; and Circle 3 at a normal retinal site, sparing the large retinal vessels (Fig. 2b, d). The positions of the circles were determined manually by comparing the fundus photographs and the LSFG color map images (Figs. 1b and 2b, d). The MBR, a quantitative index of relative blood flow velocity, was calculated in each circle using LSFG Analyzer software (v 3.0.47; Softcare). The software automatically set each circle at the same site where a previous circle had been set at baseline during follow-up. To evaluate changes in average MBR, which is a quantitative index of the “relative” blood flow velocity, the changing rates of average MBR against the initial baseline values (defined as 100%) were utilized, as previously described [16–20].

As previously demonstrated, within a certain range, the relationship between choroidal blood flow and ocular perfusion pressure (OPP) is bilinear in healthy subjects with normal eyes [21]. To exclude the possibility of such physiological responses from the results, the patient’s blood pressure and IOP were measured to calculate the OPP. Mean blood pressure (BPm) was calculated from systolic blood pressure (BPs) and diastolic blood pressure (BPd), according to the following equation: BPm = BPd + 1/3(BPs - BPd). OPP was calculated using the following equation: OPP = 2/3 BPm - IOP.

EDI-OCT measurements were obtained from the affected eye at the initial visit and 3 and 6 months after the initial visit and the fellow eye at the initial visit and 21 months after the initial visit. Choroidal thickness was determined by manually measuring the distance from the outer border of the hyper-reflective line which corresponded to the RPE to the outer border of the choroid (Fig. 2a, c), using a horizontal scan through the fovea (scan length, 12.0 mm). The choroidal thicknesses were measured at the identical sites as Circles 1–3 set in Fig. 2b (fovea, lesion site, normal retinal site, respectively) for the affected eye (Fig. 2a, c) and at the identical sites as defined Circles in the affected eye for the fellow eye. Two authors (YI, YH), blinded to clinical information, independently evaluated EDI-OCT images and the average values of two authors were compared.

In the affected eye, the decrease in MBR on the LSFG color map was clearly visualized at the initial visit and corresponded to the chorioretinal lesion (Fig. 2b). The average MBR values were as follows: 9.0 ± 0.2, 11.0 ± 0.2, and 10.0 ± 0.7 in Circle 1; 2.6 ± 0.1, 3.2 ± 0.0, and 3.4 ± 0.3 in Circle 2; 12.8 ± 0.3, 14.2 ± 0.2, and 12.9 ± 1.1 in Circle 3 at baseline and 3 and 6 months after baseline, respectively. When the changing rates in MBR were compared with the MBR levels at the initial visit (100%), 22.2 and 11.1% increments were detected in Circle 1 (the fovea) at 3 and 6 months, respectively (Fig. 2b, d, f). In Circle 2 (the lesion site), similarly, 23.0 and 30.7% increments were noted at 3 and 6 months, respectively. In contrast, the MBR in Circle 3 (normal retinal site) showed little or no change with 10.9% and 0.7% at 3 and 6 months, respectively.

In the fellow eye, the MBR at the fovea was 10.3 ± 0.4 and 10.6 ± 0.6 at baseline and 11 months, respectively and showed little change with a 2.9% increase at 11 months.

OPP was unaltered throughout the course of the disease, with values of 29.3, 33.1, and 35.1 mmHg measured at the initial visit and 3 and 6 months after the initial visit in the affected eye, respectively. In the fellow eye, OPP was 26.3 and 31.1 mmHg at the initial visit and 11 months after the initial visit, respectively.

Changes to the choroidal thickness in the affected eye are shown in Fig. 2e. Choroidal thickness in the fovea gradually increased from 284.0 ± 0.0 μm at baseline to 325.5 ± 8.5 and 317.0 ± 4.0 μm at 3 and 6 months after baseline, respectively (Fig. 2a, c, e). Similarly, choroidal thickness at the lesion site increased from 158.0 ± 2.0 μm at baseline to 171.0 ± 2.0 and 175.0 ± 2.0 μm at 3 and 6 months, respectively. In contrast, the thickness at the normal retinal site was not altered, with values of 311.0 ± 2.0, 319.0 ± 2.0, and 311.0 ± 2.0 μm at baseline and 3 and 6 months after baseline, respectively.

In the fellow eye, the thickness examined at the same sites as Circles 1–3 set in affected eye were 321.5 ± 4.5, 158.0 ± 2.0, and 373.0 ± 2.0 μm, respectively, at baseline and 311.0 ± 2.0, 144.0 ± 4.0, and 369.0 ± 2.0 μm, respectively, at 21 months after baseline. When the values at baseline were compared between the affected and fellow eyes, thickness in the affected eye was thinner than in the fellow eye except for Circle 2.

## Conclusions

In the present study, we quantitatively evaluated changes in choroidal blood flow velocity and choroidal thickness in the macular area using LSFG and EDI-OCT in a patient with ocular blunt trauma-associated chorioretinopathy. In the affected eye, the MBR was decreased at the site of the lesion at the initial visit. Moreover, the MBR and choroidal thickness during the 6-month follow-up period after baseline measurements increased by 11–22% and 33–42 μm at the fovea, and 23–31% and 13–17 μm at the lesion site, respectively; improvements in the BCVA and foveal outer retinal morphology were also observed. In contrast, these parameters showed little or no changes at the normal retinal site in the affected eye (1–11% and 0–8 μm) and the fovea in the fellow eye (3% and 11 μm). Since the OPP remained unchanged during the follow-up, the MBR changes at the lesion site during the follow-up in this patient were due to changes in choroidal blood flow velocity but not systemic hemodynamics. Considered together, our data revealed that both choroidal blood flow velocity and thickness at the lesion site increased with regression of the chorioretinopathy following blunt ocular trauma.

In the present case, the MBR at the affected area decreased in the acute stage. Experimental studies in rabbits whereby retinal opacity was induced following blunt trauma revealed that the short posterior ciliary artery, which is located in the choroidal outer layer nearest to the sclera, was broken down and flow to the periphery was affected beneath the site with retinal opacity [10, 11]. This suggests that a disruption in circulation of the large choroidal vessels beneath the lesion site plays a role in the pathogenesis by causing outer retinal damage in this disease. LSFG predominantly measures blood flow velocity in the deeper choroidal layers rather than in the choriocapillaris [22]. Therefore, our results showed that a decrease in the MBR during the acute stage might support the observations of previous experimental studies mentioned earlier.

In our case, both the MBR and choroidal thickness at the lesion site gradually increased accompanied by improvements in visual function and the outer retinal morphology. In studies that ligated the short posterior ciliary arteries of monkeys, almost all of the choriocapillaris and choroidal middle or large vessels at the ligated area were occluded 1 week after ligation [23]. However, the vascular luminal structures in the choroidal stroma were observed in spots 1 month after ligation [24]. These observations suggest that a part of the middle or large choroidal vessels at an area of infarction were recanalized after the onset of choroidal infarction. Similarly, our results might have been due to recanalization of the impaired choroidal vessels.

In choroiditis such as Vogt–Koyanagi–Harada disease and serpiginous choroiditis, our previous observations using LSFG and EDI-OCT revealed that macular choroidal blood flow velocity decreased and choroidal thickness increased during the acute stage and systemic corticosteroid therapy reversed these trends [18, 25]. Changes in the blood flow and thickness may be manifestations of a process with an “inflammatory” pattern occurring in the choroid [18, 25]. Similarly, these changes have been also observed in unilateral acute idiopathic maculopathy [20], acute posterior multifocal placoid pigment epitheliopathy [26], and acute zonal occult outer retinopathy complex [17, 19, 27–30]. In contrast, both the blood flow velocity and thickness increased in the acute stage of central serous chorioretinopathy [16, 31], which involves a sympathetic or adrenergic etiology. These changes may involve a sympathetic pattern. Interestingly, the results shown in the present case (decrease of both blood flow and thickness in the acute stage) were inconsistent with patterns described above and may be newly termed as an “vaso-occlusive” pattern in the choroid. Thus, it may be useful to evaluate the activity of choroidal involvement in patients with blunt ocular trauma-associated chorioretinopathy, using LSFG and EDI-OCT.

In the present case of chorioretinopathy associated with ocular blunt trauma, finally, LSFG and EDI-OCT results revealed that both choroidal blood flow velocity and thickness at the lesion site decreased in the acute stage and subsequently increased over time; additionally, improvements in visual function and outer retinal morphology were observed. These results suggest that LSFG and EDI-OCT are useful indices for non-invasive evaluation of the activity of choroidal involvement in this disease. Further studies with a larger number of cases are needed to establish usefulness of the MBR and choroidal thickness as indices in this disease.

## Abbreviations

BCVA: Best-corrected visual acuity
EDI-OCT: Enhanced depth imaging optical coherence tomography
ICGA: Indocyanine green angiography
LSFG: Laser speckle flowgraphy
MBR: Mean blur rate
OPP: Ocular perfusion pressure
RPE: Retinal pigment epithelium
